# Supplementary material for: Prevalence and determinants of non-communicable diseases risk factors among reproductive aged women of Nepal: Results from Nepal Demographic Health Survey 2016
Source: PLoS One. 2020 Mar 16;15(3):e0218840. doi: 10.1371/journal.pone.0218840 (PMC7075700; doi:10.1371/journal.pone.0218840)
Supplement: Attachment — Submitted filename: Zaman2015_Article_ClusteringOfNon-communicableDi.pdf [file pone.0218840.s003.pdf]

Zaman et al. BMC Public Health (2015) 15:659
DOI 10.1186/s12889-015-1938-4

BMC Public Health

RESEARCH ARTICLE Open Access

Clustering of non-communicable diseases risk factors in Bangladeshi adults: An analysis of STEPS survey 2013

M. Mostafa Zaman[1*], Mahfuzur Rahman Bhuiyan[1†], Md. Nazmul Karim[1†], MoniruzZaman[1], Md. Mukhlesur Rahman[2], Abdul Waheed Akanda[2] and Thushara Fernando[1,3]

Abstract

Background: Non-communicable diseases (NCDs) have already become major killers in Bangladesh. Once NCDs are developed, they become chronic health and economic problems. Their primary prevention is linked to their common risk factors. This study was conducted to determine the prevalence of NCD risk factors with a focus on their clustering in Bangladeshi adults.

Methods: This nationally representative study was done in 4,073 (1,812 men and 2,261 women) adults aged 25 years or older selected from rural and urban households. Multistage cluster sampling design was used. Selected variables were in line with steps I and II of WHO stepwise surveillance except alcohol.

Results: Forty-four percent used tobacco in any form. Almost 93 % did not consume adequate fruit and vegetables (5 servings or more). Thirty eight percent had low physical activity level (<600 MET-minutes/week). One-quarter (26 %) were overweight (body mass index > =25 kg/m^2). Twenty-one percent had hypertension (blood pressure > =140/ 90 mmHg or medication) and about 5 % had documented diabetes.
Upon examination of risk factor clustering, we observed that 38 % had at least three risk factors. After this threshold, clustering suddenly dropped down to a fairly low level. Using this threshold as a cut-off, clustering of risk factors was associated with age, male gender, urban residence, educational levels and quality of house in multivariate analysis.

Conclusion: Prevalence of NCD risk factors is fairly high in Bangladeshi adults with a tendency of clustering. If a risk factor such as hypertension is detected, a closer look for other risk factors has to be given in both at clinical and public health settings. Clustering raises risk by more than a summation of risk factors. Our findings, therefore, suggest that Bangladesh could expect a significant increase in NCDs in near future.

Background

Major non-communicable diseases (NCDs) such as cardiovascular diseases, cancer, diabetes, or chronic respiratory disease have already become major public health problems in Bangladesh. According to the Global Status Report on NCDs 2014 [1] of the World Health Organization (WHO), the estimated probability of premature deaths between ages 30 and 70 from any of the aforesaid NCDs is 17.5 %. The total number of deaths in 2012 was 277,500 due to NCDs giving rise to a death rate of 564.1 per 100,000 in males and 531.9 per 100,000 in females. Nearly half (49 %) of deaths were due to NCDs. These rates for NCDs are already in the web of increasing trend [2] concomitant to increasing trend of high blood pressure and diabetes in Bangladesh [3].

NCDs are caused by a few common risk factors: low intake of fruit and vegetables, low level of physical activity, tobacco use, harmful use of alcohol, obesity, raised blood pressure, raised blood cholesterol and glucose. These risk factors are commoner in prevalence and easier to detect compared to NCDs. Today's risk factors will mature to NCDs tomorrow. Their control is less expensive than treatment of full-blown NCDs. Therefore risk factor approach for NCD prevention has become popular. It is well known that NCDs are multi-factorial

* Correspondence: zamanm@who.int
†Equal contributors
[1]Division of NCD, World Health Organization, Dhaka, Bangladesh
Full list of author information is available at the end of the article

BioMed Central

© 2015 Zaman et al. This is an Open Access article distributed under the terms of the Creative Commons Attribution License (http://creativecommons.org/licenses/by/4.0), which permits unrestricted use, distribution, and reproduction in any medium, provided the original work is properly credited. The Creative Commons Public Domain Dedication waiver (http://creativecommons.org/publicdomain/zero/1.0/) applies to the data made available in this article, unless otherwise stated.

diseases. Usually more than one factors work together for early maturation of NCDs.

There have been reports from the United States [4], Brazil [5], and Indian [6] population on clustering of risk factors especially in hypertensive patients. Clustering is observable even in children [7]. There has been a clue that Bangladeshi people have a higher prevalence of clustering of NCD risk factors [8–11], This clustering phenomenon may predispose them to a higher burden of NCDs compared to populations with lower tendency of clustering. However, systematic analysis of clustering of all major risk factors is almost lacking to claim national representation for Bangladeshi people. We have conducted this study, as a periodic STEPS (STEPwise Surveillance) survey [12], to determine the prevalence of NCD risk factors with a focus on their clustering in Bangladeshi adults.

Methods

This survey was conducted in 2013 as per step-I and step-II of STEPS approach devised by the WHO [12]. The target population for this survey included all men and women aged 25 years or older living in urban and rural areas of Bangladesh.

Sampling

Sample was drawn from 21 rural villages (three from each division), seven municipalities located at upazila headquarters (one from each division as moderately urbanized area) and three city corporation areas (most urbanized area) from all seven divisions of the Country. Half of the households were identified as male and second half as female to ensure a gender balance. All eligible members were entered in a roster for identification of one per household using Kish table [13]. Using the prevalence of hypertension (18 %) among the population, and 3 % margin of error, the minimum sample size was 650. This study was proposing national estimate in four groups according to gender and urban–rural area of residence. Therefore a minimum of 2600 respondents were needed. To address the design effect (1.5) and potential response rate (80 %) it was further inflated to a final size of 4875.

Survey instrument

An adapted (mostly for socio-demographic background) questionnaire for this survey was developed using step-I and step-II of STEPS as was done in 2010 survey in Bangladesh [14]. All the core questions except alcohol (because of extremely low prevalence and cultural reasons) were used. Household information was adapted to local situation such as dwelling house's roof materials. The individual component included questions on tobacco, physical activity, and fruit and vegetable intake. Information were also obtained on treatment of hypertension and diabetes. Physical measurements were on height, weight and blood pressure. The questionnaire was in Bangla which was pretested in the field before actual survey. The questionnaire was administered by interviewers and no proxy interview was allowed.

Data collection

The field team underwent a 3-day training before deployment. Data were collected in the first quarter of 2013. Assistance from local health authority was sought to ensure proper identification of sampling unit boundaries and cooperation of the local community. After identification of eligible subjects, no sample substitution was allowed. Each field team consisted of two experienced health workers and one supervisor. Methods for ascertainment of key variables were same as described in STEPS 2010 report [15] but a brief description is given below:

Information of tobacco was collected for both smoking and smokeless forms. Those who smoked or used smokeless tobacco daily in the past 30 days were considered as 'current' user. Serving sizes of fruit and vegetables in a typical week were determined using show-cards or measuring cups (in case of cooked items). Data on time spent on moderate and vigorous physical activities (during work, leisure time and commutation) were transformed into minutes per week. They were then converted to metabolic equivalent task (MET)-minutes per week (one minute in moderate and transport related activities equal to 4 MET-minutes and one minute in vigorous activities equal to 8 MET-minutes). Information on treatment of hypertension and diabetes was sought and prescriptions (or medicine strips) were checked if necessary. Shoes and heavy clothing were removed before measuring height (to nearest cm) and weight (to nearest 0.2 kg). Blood pressure was measured using ordinary aneroid sphygmomanometers on the left arm while the participants were in sitting position after having a rest for about 5 min. Korotkoff phase V was taken as diastolic blood pressure. Mean of two readings taken two minutes apart was used in the statistical analysis.

Data analysis

Complex survey data analysis was performed to obtain population prevalence or mid-point estimates and their dispersions. Student's t test or Wilcoxon test were used, as appropriate, to compare levels in men and women. Prevalence sex-groups and association between categorical variables were examined and compared using Chi-square test. Prevalence data were adjusted to the WHO world population [16]. Multiple logistic regression analysis was done to obtain odds ratios and their confidence intervals for clustering of three or more risk

factors. All analyses were done using Statistical Package for the Social Sciences version 16.0.

Ethical considerations

This survey was done as a part of the service oriented intervention programme developing model jurisdictions. Ethical clearance was given by the Bureau of Health Education authority. Community leaders' orientations were done in all areas before starting the survey. Their consents were obtained first. In the next step verbal consent from individual respondents were obtained.

Results

We have targeted 4875 individuals but finally 4073 participated. The response rate was 83.5 % (men: 74.3 % and women: 92.7 %). We observed that, in general, NCD risk factors are highly prevalent in Bangladeshi adult population. There was hardly anyone without a risk factor and a substantial proportion of people were living with multiple risk factors.

Socioeconomic background

Of the total respondents, 44.5 % were men (Table 1). A little less than one-third (28.5 %) of the participants were from urban areas as stipulated in the survey design. Their mean age was 43 years (men: 45 and women: 41). Four in ten of them had primary education (one to five years of schooling). One-fifth of men was farmers, another one-fifth was labourers (agriculture, industrial or otherwise), and one-fifth was salary men. However, 71 %

Table 1 Sociodemographic background of the Bangladeshi adult respondents for STEPS survey 2013

			Men and women	Men	Women	P values
			n = 4073	*n* = 1812	*n* = 2261	
Age						
	Categories, n (%)					
		25–34	1287 (31.6)	468 (25.8)	819 (36.2)	
		35–44	1081 (26.5)	442 (24.4)	639 (28.3)	
		45–54	806 (19.8)	418 (23.1)	388 (17.2)	
		55–64	503 (12.3)	259 (14.3)	244 (10.8)	
		65 +	396 (9.7)	225 (12.4)	171 (7.6)	0.00
	Mean (standard deviation)		42.9 (14.1)	45.3 (14.6)	41.0 (13.4)	0.00
Residence, %						
	Urban		28.5	28.4	28.6	
	Rural		71.5	71.6	71.4	0.89
Education						
	Median years of schooling (IQ range)*		5 (0–8)	5 (1–8)	4 (0–7)	0.00
	Categories, %					
		No schooling	26.6	24.8	28.1	
		Any primary	40.0	36.7	42.7	
		Any secondary	25.5	26.4	24.8	
		Above secondary	7.8	12.1	4.4	0.00
Roof of the house made of, %						
	Cement, concrete or tiles		16.0	19.1	13.5	
	Tin sheets		59.6	57.3	61.5	
	Bamboo, thatches, straw, shacks etc.		24.4	23.6	25.0	0.00
Primary occupation, %						
	Home maker		41.0	3.9	70.7	
	Day labourer		8.4	17.2	1.4	
	Farmer		9.3	18.4	2.1	
	Business		14.3	27.5	3.6	
	Salaried staff		16.2	21.5	11.9	
	Students and others		10.8	11.4	10.3	0.00

*IQ indicates inter-quartile

of women were home makers and 12 % were salaried staff. As an index of living condition, we collected data on the construction materials of the dwelling house. Roof of six out of 10 houses were made of tins, followed by poorer quality roofs made of bamboo, thatches etc. (24 %). Only 16 % had cement or concrete roofs. These socioeconomic backgrounds are typical in Bangladesh in current days.

Distribution and prevalence of risk factors

We have presented the distribution of risk factors in a quantitative manner giving mid points and dispersions in Table 2 and prevalence of risk factors in a categorical manner using standard cut-off points in Table 3.

Median per capita consumption of fruit and vegetables was 2.0 servings per day. More than 93 % of the people consumed less than recommended minimum 5 servings of fruit and/or vegetables per day. Physical activity data of three domains (work, commutation and leisure time) were combined to determine total physical activity as is conventionally done. Median physical activity was 1680 MET-minute per week. Thirty eight percent of the participants had low level of physical activity (<600 MET-minute per week). Prevalence of daily smoking in sexes combined was 17 % with a striking sex difference (men: 38 % and women: 2 %). Prevalence of non-daily smoking and ex-smoking were 3.2 % (men: 6.7 and women: 0.4) and 5.9 % (men: 11.8 % and women: 1.3 %) respectively (data not shown). Twenty-seven percent were smokeless tobacco users (men: 28 %, women 26 %). Forty-four percent used tobacco in any form (men: 58 %, women: 32 %).

Mean body mass index (BMI calculated as Kg/meter squared) was 23.0 Kg/m^2 (Table 2). One-fifth (20 %) of the respondents were overweight (BMI > =25.0–29.9) and 5 % were obese (BMI > =30.0) (Table 3). There was no significant difference between men and women. Mean systolic blood pressure was 118 mmHg and mean diastolic was 75 mmHg (Table 2). Eleven percent had physician diagnosed high blood pressure and 10 % were receiving medication for high blood pressure. Out of them two-third (66 %) had their high blood pressure controlled (<140/90). Overall, 18 % had high level of blood pressure irrespective of taking medication. Taking in to account of blood pressure level (> = 14/90) and history of medication, 21 % of the respondents were hypertensive. A little less than 5 % of the respondents had documented (self-reported) diabetes. We did not seek any information on lifestyle modification for hypertension or diabetes.

We have examined the prevalence of risk factors stratified in to rural and urban areas. All the risk factors were persistently more prevalent in urban areas (Fig. 1). Although tobacco prevalence is a little high in urban area, as opposed to previous reports [15], it was not statistically significant.

Clustering of risk factors

We have examined the clustering (presence of multiple risk factors in an individual) of risk factors in our sample. Three-quarter of the respondents (76 %) had two or more and four in ten (37 %) had three or more, one in ten (12 %) four or more risk factors (Fig. 2). This clustering was increasingly prominent with increasing age. A sub-sample analysis of hypertensive subjects (data not shown) also showed more prominent clustering. Presence of three risk factors was considered as threshold for identifying a clustering phenomenon because after this threshold the rates dropped down suddenly. Association of this clustering with various socio-demographic factors was examined (Table 4). It was significantly higher with ageing (odds ratios ranging from 1.6 to 4.2), male gender (odds ratio 1.4), and urban residence (odds ratio 1.9) in univariate logistic regression analysis. The indices of economic achievement: education and housing were also associated with an indication that clustering was higher in richer people. In multiple logistic regression analysis also these findings remained almost similar when all the variables were entered simultaneously in to the regression model. The risk factors which attributed to the clustering, hypertension, low intake of fruit/vegetables and tobacco use were the commonest.

Discussion

The current study is the third national survey on NCD risk factors in Bangladesh and the first to describe the clustering phenomena in a nationally representative survey. In addition to the high prevalence of individual risk factors, clustering of 3 or more risk factors was also

Table 2 Distribution of non-communicable disease risk factors in Bangladeshi adults, STEPS 2013

Risk factors	Both sexes	Men	Women	*P* values
Fruits/ vegetables, servings/day[a]	2.0 (0.8, 3.0)	2.0 (0.9, 3.0)	2.0 (0.7, 3.0)	0.23
Physical activity, MET- minute/week[a]	1680 (0, 5400)	2400 (0, 8400)	980 (0, 4100)	0.00
Body mass index, Kg/m^2[b]	22.9 (4.1)	22.8 (3.7)	23.0 (4.4)	0.05
Systolic blood pressure, mmHg[b]	117.9 (15.9)	118.5 (14.1)	117.5 (17.2)	0.10
Diastolic blood pressure, mmHg[b]	75.3 (12.1)	75.7 (11.6)	75.1 (12.5)	0.08

[a]Median (interquartile range); [b]Mean (standard deviation); MET indicates metabolic equivalents

Table 3 Prevalence of NCD risk factors in Bangladeshi adults, STEPS 2013

Age (years)	Tobacco use			Fruits/ vegetables < 5 servings[a]	Low physical activity[b]	Overweight (BMI ≥25–29.9 Kg/m^2)	Obesity (BMI > =30 Kg/m^2)	Hypertension (BP ≥ 140/90 mmHg)[c]	Documented diabetes
	Smoking	Smokeless	Any form						
Men and women									
25–34	12.4	15.5	28.5	95.7	36.5	19.2	4.4	11.6	1.6
35–44	15.0	24.3	40.3	92.7	35.2	23.8	5.7	17.1	3.2
45–54	20.7	33.1	53.8	93.8	34.7	21.8	6.1	26.6	6.9
55–64	24.1	43.7	63.2	91.5	37.4	17.9	4.4	32.6	8.2
65 +	17.7	40.4	59.3	90.9	55.3	16.7	4.4	40.4	8.8
Total (unadjusted)	16.7	27.2	43.9	93.5	37.7	20.5	5.2	21.4	4.6
Total (adjusted)	17.1	28.7	45.8	93.3	38.6	20.3	5.1	23.1	5.0
Men									
25–34	29.5	18.6	48.1	94.4	35.7	17.7	3.6	10.0	1.1
35–44	34.4	23.5	55.9	92.8	31.7	24.2	3.4	13.6	2.9
45–54	38.5	31.6	63.4	93.5	27.3	21.8	5.3	23.9	8.1
55–64	34	42.5	71.8	89.2	29.3	18.5	4.6	30.1	9.3
65 +	29.8	37.3	60.9	89.8	53.8	13.3	4	39.6	9.8
Total (unadjusted)	34.8	28.5	58.5	92.5	34.1	19.8	4.1	20.6	5.4
Total (adjusted)	33.2	28.5	58.3	92.4	34.2	19.6	4.1	20.7	5.4
Women									
25–34	2.6	13.7	17.3	96.5	37.0	20.0	4.9	12.5	2.0
35–44	1.6	24.9	29.6	92.6	37.6	23.5	7.4	19.6	3.4
45–54	1.5	34.8	43.6	94.1	42.8	21.9	7.0	29.4	5.7
55–64	2.9	45.1	54.1	93.9	45.9	17.2	4.9	35.2	7.0
65 +	2.9	44.4	57.3	92.4	57.3	21.1	5.8	41.5	7.6
Total (unadjusted)	2.2	26.2	32.3	94.4	40.6	21.1	6.0	22.1	3.9
Total (adjusted)	2.2	29.5	36.5	94.1	42.5	21.0	6.0	25.0	4.6
P values[d]									
	0.00	0.00	0.00	0.01	0.00	0.31	0.01	0.28	0.03

[a] <400 gm/day
[b] <600 MET-minutes/week
[c]and/or medication for hypertension
[d]Men compared to women

found to be widely prevalent predisposing Bangladeshi people to a greater risk of NCDs.

Although Bangladeshi people consume vegetables every day, the quantity is low. This is not because vegetables are not always available; this is mostly because vegetables are not considered as nutritious food by many people. Seasonal fruit, sometimes grown in abundance, are not popular because people do not consider them as good fruit. Although popular, imported varieties remain unaffordable to many people leading to a very low consumption of fruit. Therefore a campaign is needed to popularize local fruit. A '5-a-day' campaign to promote 5 servings per day can be taken as was done in England. This may be exploited to reverse the existing *'misti'* (sweets full of simple sugar and saturated fat) culture in Bangladesh to a fruit culture. Fruit should no more be considered a sick person's diet but it should be eaten more frequently to prevent sickness.

The global estimate for prevalence of physical inactivity among adults is 17 % [17] whereas our observed rate is more than double (38 %). Physical inactivity problem in Bangladeshi adults cannot be generalized. There are many people in Bangladesh who endure hard physical labour for their livelihood leading to a very thin body mass. In our sample, one-quarter of respondents were rather thin (BMI < 18.5 Kg/m^2). Poorly planned urbanization is the major reason for higher level of physical inactivity in urban areas. However this is now visible even in rural areas. Junk food and mechanization of life might be major contributors. Most physical activities in our sample was from work

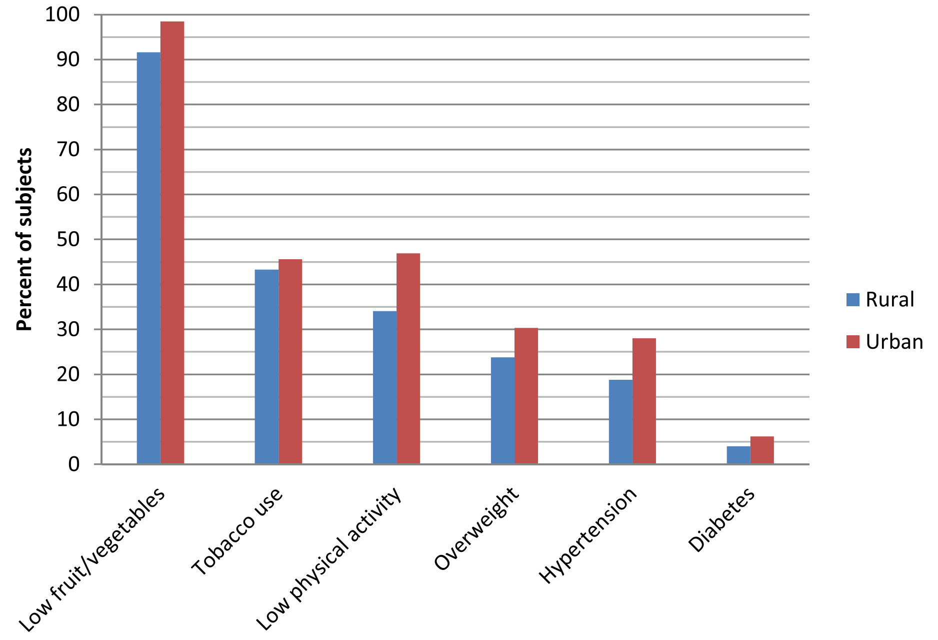

Fig. 1 Distribution of Bangladesh adults by the number of NCD risk factors*, STEPS 2013. *Risk factors include 1) current tobacco use, 2) low fruit and vegetables intake, 3) low physical activity, 4) overweight, 5) hypertension and 6) documented diabetes

or job related. Commutation contributed to some extent but leisure time physical activity was very low. Commutation related activities could be promoted by encouraging bicycles and keeping footpaths walkable. Promotion of leisure time related activities will require engagement of non-health sectors creating recreational facilities including provision of play grounds, parks, sports, etc. An innovative strategy for uplifting physical activity of women, because they had low level of physical activity, without conflicting with social and religious norms is required.

Prevalence of both smoking (cigarette, *bidi*, etc.) and smokeless tobacco (*zarda, gul, pan-masala*, etc.) use is high in men. Although magnitude of tobacco use still remains very high, we observed lower prevalence than previous national level studies (see below for trend data). We have already reported the problem of dual use of

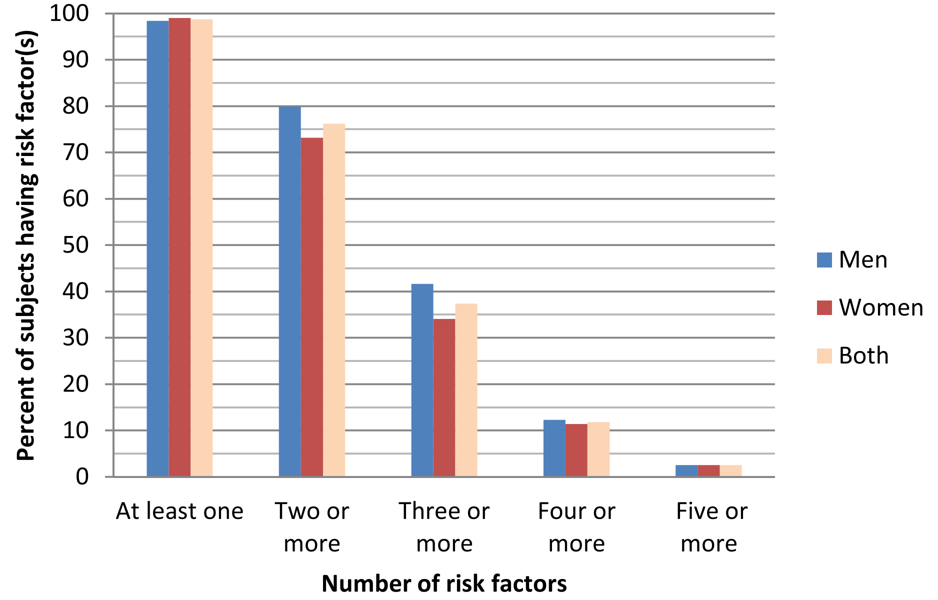

Fig. 2 Prevalence of risk factors in urban and rural areas of Bangladesh, STEPS 2013

Table 4 Association of socio-demographic factors with clustering of three or more risk factors in Bangladeshi adults, STEPS 2013

		Odds ratio (95 % confidence interval)	Adjusted odds ratio (95 % confidence interval)
Age groups			
	25–34	Reference	Reference
	35–44	1.6 (1.3–1.9)	1.7 (1.4–2.0)
	45–54	2.4 (2.0–2.9)	2.6 (2.1–3.2)
	55–64	2.7 (2.1–3.3)	3.0 (2.4–3.7)
	65+	4.2 (3.3–5.3)	4.8 (3.7–6.2)
Sex			
	Women	Reference	Reference
	Men	1.4 (1.2–1.6)	1.2 (1.0–1.4)
Residence			
	Rural	Reference	Reference
	Urban	1.9 (1.7–2.2)	2.0 (1.7–2.3)
Education			
	No education	Reference	Reference
	Any primary	1.2 (1.0–1.4)	1.3 (1.1–1.6)
	Any secondary	1.0 (0.8–1.2)	1.2 (1.1–1.6)
	Above secondary	1.2 (0.9–1.5)	1.3 (1.0–1.7)
Quality of main living house			
	Tin, bamboo, thatches, straw, shacks etc.	Reference	Reference
	Cement, concrete or tiles	1.4 (1.2–1.7)	1.2 (1.0–1.5)

tobacco in men [18]. Dual use exposes Bangladeshi men to an additional risk of killer NCDs [19] as has been observed in many other populations [20]. Unlike men, women hardly have any problem of dual use because smoking is extremely low in women. Women's smoking is considered as impolite by the society. They, on the other hand, use smokeless tobacco with betel quid which is responsible for variety of diseases such as heart diseases, stroke, oral cancer, etc. Unfortunately it was not included in the Smoking Control Act [21]. Use of smokeless tobacco as a component of betel quid has a high cultural acceptance in Bangladesh. Therefore culturally appropriate public awareness will be required. Considering the public health consequences of smokeless tobacco, the amended version (2013) of the aforesaid Act has addressed smokeless tobacco adequately. Although tax measures are visible, more stringent actions are needed to curb tobacco epidemic in addition to other measures.

Overweight and obesity have been growing in Bangladesh. Prevalence of overweight in a rural population in 1998 was 6.5 % [22]. It is 10.2 % in rural area in 2010 [15] and 20.3 % in the current study. An obese baby is still considered healthier than a lean baby in Bangladesh. A fat lady is considered prettier than a thin one. Therefore public awareness campaign is to be undertaken to counter the drive to have a fat nation.

Elevated blood pressure is a recognized intermediate risk factor in developing stroke and heart attacks. One meta-analysis of population-based hypertension studies done up to 1994 reported a prevalence of 11.3 % [23]. Another meta-analysis of subsequent population studies (published from 1995 to 2009) reported a prevalence of 13.5 % [24]. In 2010 survey [15] it was 18 %; in the current study we report here a further high prevalence of hypertension (21) %. Measures to contain increasing blood pressure are seriously needed. Dietary salt must be targeted because its intake is very high (11–17 gm per day) in Bangladeshi people [25]. In spite of being a common problem and simple to identify, hypertension detection and treatment status is far from adequate in Bangladesh. Primary health care system has a good infrastructure in Bangladesh spreading all over the country. Therefore primary health care approach needs to be used to ensure adequate detection and treatment.

The distribution of risk factors was examined for rural and urban strata. As expected, all the risk factors were more prevalent in urban areas (Fig. 1). However the higher prevalence of tobacco use in urban area (although statistically non-significant) is somewhat not supportable [15]. In a few of the villages there might have been a tobacco control intervention. Therefore this difference should be interpreted with caution.

We could not measure blood glucose in this survey. About 5 % people had documented diabetes. Measurement of glucose could presumably double the prevalence because 6.8 % prevalence in a rural area has already been reported [26]. It is understandable that in urban area it will be even higher [27]. There are lines of evidences that the prevalence of diabetes is rising in Bangladesh possibly because of recent substantial changes in lifestyle. This could reflect the effect of poorly planned urbanization that lacks in environment for physical activity, and unregulated food industries promoting junk food.

We have made a brief review of the all three STEPS surveys done (2006 [28], 2010 [15] and the current one) at national level in the history of Bangladesh. Summary findings are plotted in a bar chart (Fig. 3). There is a clear indication that low intake of fruit/vegetables, sedentary behavior, overweight, hypertension and documented diabetes have been increasing. This is not surprising in a society with increasing mechanization of life paralleled to increasing availability of junk foods in absence of any specific intervention or programme. Population ageing is not the possible answer because out prevalence data are standardized for age [16]. Fortunately we have a fairly good tobacco

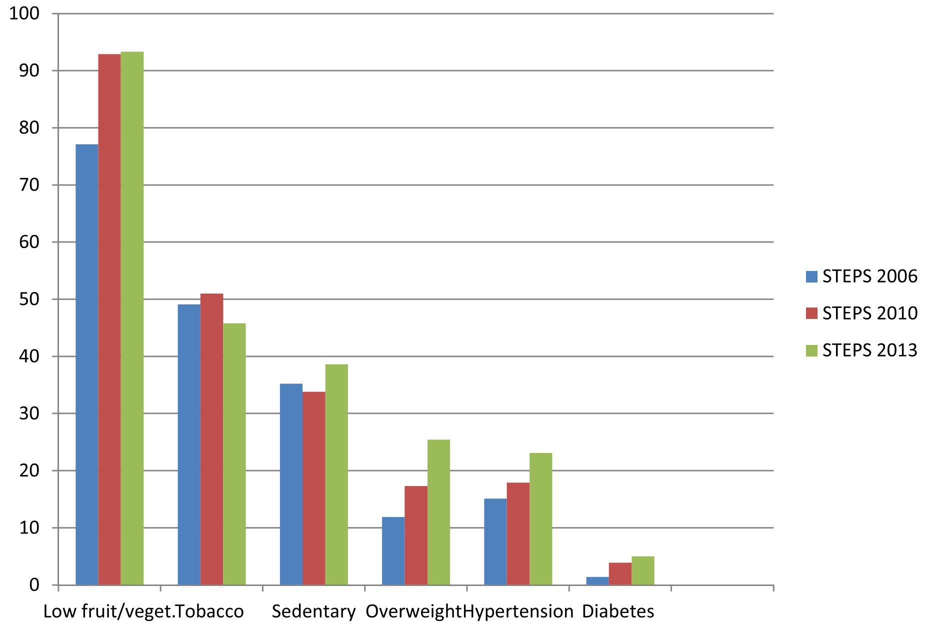

Fig. 3 Comparison of three STEPS surveys' results (2006, 2010 and 2013) in Bangladeshi adults aged 25 years or older. *Tobacco use in any form; fruit and vegetable intake, <5 servings per day; physical activity, <600 MET-minutes per week; overweight, body mass index > = 25 kg/m^2; diabetes, self-reported (documented); all are national level surveys

control programme for about two decades. Therefore an opposite trend of tobacco is observable in Bangladesh.

Presence of one risk factor in turn increases the likelihood of having other risk factors showing a clustering phenomenon. Presence of hypertension may act as a pivot for clustering to happen given that the clustering was more prominent hypertensive subjects [5]. Clustering phenomenon in our sample was prominent with increasing age. Therefore detection of hypertension at an early age can be used as an entry point for preventing clustering of risk factors in Bangladeshi people.

Our study has a few important limitations. We could not measure blood glucose but collected data on self-reported/documented diabetes. This by no means provides the real prevalence estimate of diabetes. We believe this is only half of the actual case. Estimation of blood cholesterol could be more information to describe comprehensively all major risk factors of NCDs.

Conclusion

Prevalence of NCD risk factors is fairly high in Bangladeshi adults with a tendency of clustering. If a risk factor such as hypertension is detected, a closer look for other risk factors has to be given in both at clinical and public health settings. Clustering raises risk by more than a summation of risk factors. Our findings suggest that Bangladesh could expect a significant increase in NCDs in near future. This in turn will create an increased burden to the health care services, and loss of productivity due to due to deaths and disabilities at peak working ages in case of national inactions.

Competing interest

The authors declare that they have no competing interest.

Authors' contributions

MMZ designed the study, developed the protocol, guided data analysis, interpreted the results critically, and wrote the manuscript; MRB trained enumerators, coordinated and supervised data collection, controlled quality at all stages, and analyzed the data; MNK prepared the study protocol especially the sampling, field tested it and trained the enumerators; M analyzed data and reviewed the manuscript; MMR supervised filed execution played key role in quality control; AWA reviewed the protocol critically, guided field operations and played the role of guarantor of field operations; and TF provided overall guidance and reviewed protocol and manuals critically; he is the guarantor of the study. All authors read and approved the final manuscript.

Acknowledgements

The survey was done with technical assistance the WHO Country Office for Bangladesh. Collaborators in seven divisions were: Barendra Unnyan Sangstha (Rajshahi division), Ekhlaspur Centre of Health (Chittagong division), Eco Social Development Organization (Dhaka division), LAMB Hospital (Rangpur division), MUKTI Associates (Barisal division), Nalta Hospital and Community Health Foundation (Khulna division), and Reliant Women Development Organization (Sylhet division). Mr Abdus Salam of the Bureau of Health Education supervised field activities of the Project. Mr Reazwanul Haque Khan and Ms Suraiya Akter of WHO played key roles in field operation and data management.
The authors alone are responsible for the views expressed in this article and they do not necessarily represent the views, decisions or policies of the institutions with which they are affiliated.

Author details

[1]Division of NCD, World Health Organization, Dhaka, Bangladesh. [2]Division of Training, Bureau of Health Education, Dhaka, Bangladesh. [3]Current address: Division of Planning and Management, World Health Organization Regional Office for South East Asia, New Delhi, India.

Received: 23 December 2014 Accepted: 12 June 2015
Published online: 14 July 2015

References

1. Global status report on noncommunicable diseases 2014. World Health Organization, Geneva, Switzerland, 2014. Available at http:// http://www.who.int/nmh/publications/ncd-status-report-2014/en/ [Last accessed on 11 May 2015].
2. Karar ZA, Alam N, Streatfied PK. Epidemiologic transition in rural Bangladesh, 1986–2006. Glob Health Action. 2009; doi:10.3402/gha.v2i0.1904.
3. Zaman MM. Trends in non-communicable diseases (paper presented to the National Public Health Conference, 2013) Available at: http://www.iedcr.org/pdf/files/NPHC%20WEB/UPLOAD-4/Dr.Zaman.pdf (last accessed on 29 December 2013).
4. Weycker D, Nichols GA, O'Keeffe-Rosetti M, Edelsberg J, Khan ZM, Kaura S, et al. Risk-factor clustering and cardiovascular disease risk in hypertensive patients. Am J Hypertens. 2007;20:599–607.
5. Barreto SM, Passos VMA, Firmo JOA, Guerra HL, Vidigal PG, Lima-Costa MFF. Hypertension and clustering of cardiovascular risk factors in a community in Southeast Brazil - The Bambuí Health and Ageing Study. Arq Bras Cardiol. 2001;77:576–81.
6. Ramachandran A, Snehalatha C, Latha E, Satyavani, Vijay V. Clustering of cardiovascular risk factors in urban Asian Indians. Diabetes. 1998;21:967–71.
7. Katzmarzyk PT, Srinivasan SR, Chen W, Malina RM, Bouchard C, Berenson GS. Body mass index, waist circumference, and clustering of cardiovascular disease risk factors in a biracial sample of shildren and adolescents. Pediatrics. 2004;114:e198–205. URL: http://www.pediatrics.org/cgi/content/full/114/2/e198.
8. Zaman MM, Ahmed J, Choudhury SR, Numan SM, Islam MS, Parvin K. Prevalence of metabolic syndrome in rural Bangladesh women. Diabetes Care. 2006;29:1456–7.
9. Siddique MA, Sultan MAU, Haque S, Zaman MM, Ahmed CM, Rahim MA, et al. Clustering of metabolic factors among the patients with essential hypertension. Bangladesh Med Res Counc Bull. 2008;34:71–5.
10. Jesmin S, Islam MR, Islam AMS, Mia MS, Sultana SN, Zaedi S, et al. Comprehensive assessment of metabolic syndrome among rural Bangladeshi women. BMC Public Health. 2012;12:49.
11. Khanam MA, Qiu C, Lindeboom W, Streatfield PK, Kabir ZN, Wahlin A. The metabolic syndrome: prevalence, associated factors, and impact on survival among older persons in rural Bangladesh. PLoS ONE. 2011;6:e20259. doi:10.1371/journal.pone.0020259.
12. Bonita R, de courten M, Dwyer T, Jamrozik K, Winkelmann R. Surveillance of risk factors for non-communicable diseases: The WHO STEP wise approach. Summary. Geneva: World Health Organization; 2001.
13. Kish L. A procedure for objective respondent selection with the household. J Am Stat Assoc. 1949;44:380–7.
14. Non-communicable disease risk factor survey Bangladesh 2010. World Health Organization, Dhaka, Bangladesh. Available at http://www.searo.who.int/bangladesh/publications/ncd_risk_factor_2010/en/. [Last accessed on 9 May 2015].
15. Zaman MM, Rahman MM, Rahman MR, Bhuiyan MR, Karim MN, Chowdhury MJ. Prevalence of risk factors for non-communicable diseases in Bangladesh. Indian J Public Health. (In press).
16. Ahmad OB, Boschi-Pinto C, Lopez AD, Murray CJL, Lozano R, Inoue M. Age standardization of rates: a new WHO standard, GPE Discussion Paper Series: No.31. Geneva, Switzerland: World Health Organization; 2001.
17. Mathers CD, Loncar D. Projection of global mortality and burden of disease from 2002 to 2030. PLoS Med. 2006;3:442.
18. Zaman MM, Bhuiyan MR, Huq SM, Rahman MMR, Sinha DN, Fernando T. Dual use of tobacco among Bangladeshi men. Indian J Cancer. 2014;51:S41–9. doi:10.4103/0019-509X.147481.
19. Rahman MA, Zaman MM. Smoking and smokeless tobacco consumption: Possible risk factors for coronary heart disease among young patients attending a tertiary care cardiac hospital in Bangladesh. Public Health. 2008;122:1331–8.
20. Teo KK, Ounpuu S, Hawken S, Pandey MR, Valentin V, Hunt D, et al. Tobacco use and risk of myocardial infarction in 52 countries in the INTERHEART study: A case-control study. Lancet. 2006;368:647–58.
21. Smoking and tobacco products usage (control) act, 2005. Government of the People's Republic of Bangladesh. Available at http://www.searo.who.int/tobacco/data/bangladesh_english.pdf [Last accessed on 10 May 2015].
22. Zaman MM, Yoshiike N. Prevalence of overweight defined by body mass index in a rural adult population of Bangladesh. J Health Popul Nutr. 2003;21:162–3.
23. Zaman MM, Rouf MA. Prevalence of hypertension in a Bangladeshi adult population. J Hum Hypertens. 1999;13:547–9.
24. Moniruzzaman, Taleb A, Rahman S, Acharyya A, Islam FA, Ahmed MSA, et al. Prevalence of hypertension among the Bangladeshi adult population: a meta-analysis. Regional Health Forum. 2013;17:15–9.
25. WHO. Report of the Expert Meeting on Population Sodium Reduction Strategies for Prevention and Control of Noncommunicable Diseases in the South-East Asia Region, 11–13 December 2012. New Delhi, India, WHO 2013. Available at http://www.searo.who.int/entity/noncommunicable_diseases/documents/sea_ncd_88.pdf [last accessed on 6 May 2014].
26. Rahim MA, Akhtar H, Khan AKA, Sayeed MA, Ali SMK, Vaaler S. Rising prevalence of type 2 diabetes in rural Bangladesh: A population based study. Diabetes Res Clin Pract. 2007;77:300–5.
27. Hussain A, Rahim MA, Azad Khan AK, Ali SM, Vaaler S. Type 2 diabetes in rural and urban population: diverse prevalence and associated risk factors in Bangladesh. Diabet Med. 2005;22:931–6.
28. Zaman MM, Choudhury SR, Ahmed J, Talukder MH, Rahman AHMS. Blood glucose and cholesterol levels in Bangladeshi adult population: Results from STEPS 2006 survey. Indian Heart J. (In press).

Submit your next manuscript to BioMed Central and take full advantage of:

- **Convenient online submission**
- **Thorough peer review**
- **No space constraints or color figure charges**
- **Immediate publication on acceptance**
- **Inclusion in PubMed, CAS, Scopus and Google Scholar**
- **Research which is freely available for redistribution**

Submit your manuscript at
www.biomedcentral.com/submit

BioMed Central
