# Supplementary material for: Prevalence and determinants of non-communicable diseases risk factors among reproductive aged women of Nepal: Results from Nepal Demographic Health Survey 2016
Source: PLoS One. 2020 Mar 16;15(3):e0218840. doi: 10.1371/journal.pone.0218840 (PMC7075700; doi:10.1371/journal.pone.0218840)
Supplement: Attachment — Submitted filename: PLOS ONE REVIEW.docx [file pone.0218840.s001.docx]

**Comments to the author**

**Title:** Socio demographic correlates and clustering of non-communicable disease risk factors among reproductive aged women in Nepal: Results from the Nepal Demographic Health Survey.

**General comments:** The research article addresses a very important topic within the context of a developing country experiencing epidemiological transition. Clustering of NCD risk factors predisposes women to the risk of developing NCDs. The article is poorly written but with much improvement it has the potential to be published. There is need to consider language usage, thus the authors should not use colloquial language but statements should be supported by references to enhance authenticity of claims. The discussion section should be concise and provide reasons for any differences in findings when compared to other studies elsewhere within similar context. The authors have not followed the journal’s referencing style.

**Abstract**

The structure of the abstract does not follow the journal requirements. The abstract needs to be structured into the following;

- Background
- Methods
- Results
- Conclusion

Page 2 Line 31: The authors have inappropriately used the word similarly.

Page 2 line 36: Dalit) - correction has to be made.

Page 2 line 39: In the conclusion section the authors indicate that the clustering of NCD risk factors was more vulnerable groups such as widow/separated, Dalit and **Janajati** although for Janajati they have not shown prevalence ratios. Again there are two full stops (..).Correct that.

**Introduction**

Check on the referencing style…the reference number come before a full stop (period), not after.

Page 3 Line 50, 51: There is textual overlap with previously published work on the sentence; …. *NCDs share the common risk factors such as low intake of fruit and vegetables, low level of physical activity, tobacco use, harmful use of alcohol, obesity, raised blood pressure, raised blood cholesterol and glucose.* As a result there is need for citation and paraphrasing.

Page 3 Lines 53-60 there is need for language editing. For instance the sentence,… *In context of Nepal, STEPS survey 2013, reported that 15.5% of general population and 11.4% of women had three or more risk factors of NCD in them* Can be corrected to read as *In Nepal, the STEPS survey 2013 indicated that 15.5% in the general population and 11.4% of women reported three or more risk factors for NCDs.*

Page 3 Line 61-62: There is need for citation for this statement.

The general observation is that there is need to consider language usage, thus the authors should not use colloquial language but statements should be supported by references to enhance authenticity of claims. This section can be improved.

**Methodology**

The methodology for the study is well explained. Perhaps a question one would ask is that since the NDHS used a multi stage stratified sampling, how were cluster and sample design effects dealt with in the analysis of data.

**Results**

Page 12, Line 110: Make a correction to the first sentence ‘Just over half (53.95%) of the participants were of aged 15-29 years, the highlighted word should be ages. Make similar corrections across the result section and ensure that proper language is used for interpretation of results.

Page 14, Line 121: 26.08%, you can’t stand the sentence with a number. Kindly make correction. Moreover its ‘one risk factor’ Not ‘One risk factors’. There is need for language editing to remove typing and grammatical errors.

There is an over-use and at times misuse of the word ‘similarly’.

For interpretation of table 3, insert confidence intervals for the adjusted prevalence ratios (APR) e.g page 24 line 155, put in confidence intervals together with APRs for primary, secondary or higher education).

Under the subsection **‘Overweight’** page 24, Line 164-170 there are many typos, please make language corrections.

**Discussion**

Page 26 line 201-201 the sentence is not clear….*So, this study aim to identify at risk women to possess NCD risk factors*…. You need to rephrase the sentence.

There is no need to categorise the discussion into sub headings. The authors need only to discuss key findings, what are unique and important findings of the study. Only discuss such…what is new and emerging? There are six pages on the discussion section; it shows lack of focus and discussion of salient issues. The discussion section should be concise and provide reasons for any differences in findings when compared to other countries. There should be a subsection on strengths and limitations of the study.

**References**

The authors have not followed the journal’s referencing style. They use the APA style in the reference section, while in the text they use AMA.
