# Supplementary material for: Prevalence and determinants of non-communicable diseases risk factors among reproductive aged women of Nepal: Results from Nepal Demographic Health Survey 2016
Source: PLoS One. 2020 Mar 16;15(3):e0218840. doi: 10.1371/journal.pone.0218840 (PMC7075700; doi:10.1371/journal.pone.0218840)
Supplement: Attachment — Submitted filename: PONE_reviewer.docx [file pone.0218840.s002.docx]

**Socio-demographic correlates and clustering of Non-Communicable Diseases risk factors among reproductive aged women of Nepal: Results from Nepal Demographic Health Survey 2016**

Investigating the clustering of non-communicable diseases risk factors is important in the wake of NCDs epidemic in low and middle income countries. The strength of this study is its national representativeness. However, I doubt if there is sufficient data to explore clustering in this study population. Whereas, there are 8 common NCD risk factors – Smoking, Alcohol intake, Unhealthy diet and physical inactivity (behavioural factors), obesity, hypertension, hyperglycaemia and hyperlipidaemia (biological factors). Authors explored just three of these factors they also did not clearly define what clustering is in their study. Although, the research has merit, the paper is poorly written as it is.

Check this - Zaman et al. Clustering of non-communicable diseases risk factors in Bangladeshi adults: An analysis of STEPS survey 2013. BMC Public Health (2015) 15:659 DOI 10.1186/s12889-015-1938-4

Title: Title is not appropriate as it is, authors should do further analysis or change the title.

Background: Fair – Can be reworked e.g. lines 62-63 “Thus to tackle with NCDs, the best strategy is to identify and modify the behavioural risk factors that causes NCDs” is not in tandem with the general objective of the work.

Methodology: Most poorly documented part of the manuscript. Authors needto follow the systematic reporting of Plos one articles. There are several articles published on NCD risk factors

1. No mention is made of the data collection instrument – was it the stepwise instrument which will allow for comparison with other studies.
2. Variable definitions of outcome variablee not precise e.g. lime 90-91 *Current tobacco use includes either daily or occasional smoking or use of smokeless tobacco (snuff by mouth, snuff by nose, chewing tobacco and betel quid with tobacco)*
3. Anthropometric measures
4. Biological measures
5. Data processing – Very little information
6. Data Analysis – Not clear

Results

a. Table 1 should be shortened Provinces not likely to be meaningful to an international audience.
b. Reformat Table 2
c. Why was prevalence ratios used and not odds ratios, but were reported as odds ratios “more likely”
d. Multivariate of Clustering Risks should be on a separate table – preferably present – mean risk factors. Incidente ratios and robust Standard errors

Discussion not acceptable in this current format

Ikeola Adeoye
