# Supplementary material for: Prevalence and determinants of non-communicable diseases risk factors among reproductive aged women of Nepal: Results from Nepal Demographic Health Survey 2016
Source: PLoS One. 2020 Mar 16;15(3):e0218840. doi: 10.1371/journal.pone.0218840 (PMC7075700; doi:10.1371/journal.pone.0218840)
Supplement: Attachment — Submitted filename: PONE_reviewer two.doc [file pone.0218840.s004.doc]

**Socio-demographic correlates and clustering of Non-Communicable Diseases risk factors among reproductive aged women of Nepal: Results from Nepal Demographic Health Survey 2016**

Investigating the clustering of non-communicable diseases risk factors is important in the wake of NCDs epidemic in low and middle income countries. The strength of this study is its national representativeness. However, I doubt if there is sufficient data to explore clustering in this study population. Whereas, there are 8 common NCD risk factors – Smoking, Alcohol intake, Unhealthy diet and physical inactivity (behavioural factors), obesity, hypertension, hyperglycaemia and hyperlipidaemia (biological factors). Authors explored just three of these factors they also did not clearly define what clustering is in their study. Although, the research has merit, the paper is poorly written as it is.

Check this - Zaman et al. Clustering of non-communicable diseases risk factors in Bangladeshi adults: An analysis of STEPS survey 2013. BMC Public Health (2015) 15:659 DOI 10.1186/s12889-015-1938-4

Title: Title is not appropriate as it is, authors should do further analysis or change the title.

***Response****: Thank you for your very informative suggestion. Manuscript has attempted to provide NCDs related risk factors details on reference to socio-demographic factors. Along with that, manuscript has also attempted to include clustering of NCD risk factors which are available on Demographic and Health survey. So, we believe present title justify the findings presented. However, if reviewer suggests any appropriate alternative topics, it can be considered.*

Background: Fair – Can be reworked e.g. lines 62-63 “Thus to tackle with NCDs, the best strategy is to identify and modify the behavioural risk factors that causes NCDs” is not in tandem with the general objective of the work.

*Response: It has been rephrased aligning with objective of study*.

Methodology: Most poorly documented part of the manuscript. Authors needto follow the systematic reporting of Plos one articles. There are several articles published on NCD risk factors

1. No mention is made of the data collection instrument – was it the stepwise instrument which will allow for comparison with other studies.

***Response:*** *Methodology section has been revised. Details has been mentioned. Regarding data collection instrument, since it is Demographic and Health Survey, it has got its own universally standardized data collection tools. So, it is different than that of STEPwise instrument.*

2. Variable definitions of outcome variable not precise e.g. lime 90-91 *Current tobacco use includes either daily or occasional smoking or use of smokeless tobacco (snuff by mouth, snuff by nose, chewing tobacco and betel quid with tobacco)*

***Response****: Thank you for suggestion. Definition used here is adopted from table 3.13 of NDHS 2016 .*

Anthropometric measures

*Response: Regarding anthropometric measures, BP measurement details have been provided on manuscript. Regarding weight and height, it has been measured by following universally standardized process and procedure. Details are mentioned on DHS Biomarker Field manual that has been mentioned reference list* Biological measures

***Response****: No biological measures have been considered for this manuscript.*

Data processing – Very little information

*Response: Details of the data processing have been elaborated in the main report that has been cited in the manuscript. We considered writing specific parts of analysis relating to this manuscript to limit the length of manuscript.*

3. Data Analysis – Not clear

***Response:*** *Complex survey analysis on stata 15.1 was carried out considering Taylor linearization method of calculation Standard error. Descriptive, bivariate analysis was done to assess relationship. For multivariable analysis multiple poisson regression was considered using APR (adjusted prevalence ratio)*

Results

a. Table 1 should be shortened Provinces not likely to be meaningful to an international audience.

***Response****: Nepal has recently undergone restructuring process moving from unitary to federal structure. Although seven provinces were created by constituent assembly they are yet to be named (only three out of seven are named officially) and referred in official documents with number and we adopted the same in our study. Due to recent restricting process, Nepal lacks information on province wise fashion. So, province wise information may be useful for national as well international policy maker and decision to re-design the program related with NCDs.*

b. Reformat Table 2

***Response****: it has been reformatted.*

c. Why was prevalence ratios used and not odds ratios, but were reported as odds ratios “more likely”

***Response****: Considering interpretation easiness, prevalence ratio is more interpretable and easier to communicate to non-specialists than the odds ratio. Several studies have also recommended the use of prevalence ratio. Reference documents are available here:*

- **Alternatives for logistic regression in cross-sectional studies: an empirical comparison of models that directly estimate the prevalence ratio.**

[Barros AJ](https://www.ncbi.nlm.nih.gov/pubmed/?term=Barros AJ%5BAuthor%5D&cauthor=true&cauthor_uid=14567763)^1^, [Hirakata VN](https://www.ncbi.nlm.nih.gov/pubmed/?term=Hirakata VN%5BAuthor%5D&cauthor=true&cauthor_uid=14567763). (link: <https://www.ncbi.nlm.nih.gov/pubmed/14567763>)

- **The Burden and Determinants of Non Communicable Diseases Risk Factors in Nepal: Findings from a Nationwide STEPS Survey**

Krishna Kumar Aryal  ,Suresh Mehata ,Sushhama Neupane et.al (link: [https://journals.plos.org/plosone/article?id=10.1371/journal.pone.0134834#pone.0134834.ref019](https://journals.plos.org/plosone/article?id=10.1371/journal.pone.0134834#pone.0134834.ref019))

d. Multivariate of Clustering Risks should be on a separate table – preferably present – mean risk factors. Incidente ratios and robust Standard errors

***Response***: *Thank you for suggestion. Table has been reformatted.*

Discussion not acceptable in this current format.

*Response: Discussion chapter has been reformatted*

Ikeola Adeoye
