# Supplementary material for: Prevalence and determinants of non-communicable diseases risk factors among reproductive aged women of Nepal: Results from Nepal Demographic Health Survey 2016
Source: PLoS One. 2020 Mar 16;15(3):e0218840. doi: 10.1371/journal.pone.0218840 (PMC7075700; doi:10.1371/journal.pone.0218840)
Supplement: Attachment — Submitted filename: Manuscript Review 2.docx [file pone.0218840.s005.docx]

**Manuscript Review 2**

**Title:** Socio-demographic correlates and clustering of Non-Communicable Diseases risk factors among reproductive aged women of Nepal: Results from Nepal Demographic Health Survey 2016

**Manuscript Number:** PONE-D-19-15867R1

**General Comments**

The revised manuscript looks improved in many ways. The authors have addressed many comments which were raised in the previous review. There is general flow of ideas from the introduction to the conclusion section. Consequently this is a much improved version of the manuscript. Most of the sections are improved. However there are minor comments which authors need to address before the manuscript can be accepted for publication. Moreover, there is need for language editing.

**Introduction section**

Line 61-at the end of the reference and beginning of the sentence which starts with ‘*Evidence show*…..’ there is need for spacing

Line 64-The entire sentence need to be reconsidered, something is missing, either a conjunctive ‘and’ or a comma before 11.4% of

Line 65-The word *‘indicative’* should be replaced by *‘indication’*

Line 70- The word ‘has’ needs to be replaced with ‘have’ since women is plural

Line 72-74-The sentence needs to be rephrased to read better. The word *determinates* is supposed to be *determinants*.

**Methodology**

For me this is section has been well revised and is well presented

**Results**

Tables look more organized now, and the section is easy to follow. Meanwhile there should be consistency in interpretation of results, for instance you cannot say slightly over one third of women reported multiple NCD risk factors, while only 6.3% reported a single NCD risk factor. These are two different ways of interpreting results in the same sentence and should be avoided. Choose one and stick to it for consistency.

For interpretation of results in table 3 and 4, the Adjusted Prevalence Ratios & Adjusted Risk Ratios are supposed to be in brackets to put emphasis on the interpretation. For instance, in lines 189-191 the sentence is *‘Compared to the women with no education, women who had pursued secondary level of education were (ARR: 0.87; 95% CI:0.77-0.98) times less likely to experience NCD risk factors’*

The correct and the conventional way of writing this sentence is;

*‘Compared to the women with no education, women who had pursued secondary level of education were less likely (ARR: 0.87; 95% CI:0.77-0.98) to experience NCD risk factors.’*

Consider making the correction.

**Discussion**

This part is well written. However there are some minor comments noted.

Line 208- replace 3 fold with three-fold

Line 235-cf? Make a correction

**References**

No comments, they follow the journal style.
