# Supplementary material for: Prevalence and determinants of non-communicable diseases risk factors among reproductive aged women of Nepal: Results from Nepal Demographic Health Survey 2016
Source: PLoS One. 2020 Mar 16;15(3):e0218840. doi: 10.1371/journal.pone.0218840 (PMC7075700; doi:10.1371/journal.pone.0218840)
Supplement: Attachment — Submitted filename: PONE_reviewer_Nepal 2nd.docx [file pone.0218840.s006.docx]

**Socio-demographic correlates and clustering of Non-Communicable Diseases risk factors among reproductive aged women of Nepal: Results from Nepal Demographic Health Survey 2016 – Second Review**

The manuscript is much improved as the authors have addressed many of the initial concerns. However, the authors still need to address a few more major concerns.

1. The CHOICE of the DATA utilized for their study.	Why did the authors use the Nepal Demographic Health Survey 2016 which does not directly address NCD risk factors when Nepal has conducted the stepwise SURVEY which addresses? All the 8 NCD risk factors which enables a more robust exploration of NCD risk factors than the DHS data.

Researchers have investigated prevalence and factors of NCD in Nepal using the steps SURVEY.

i. Aryal KK, Mehata S, Neupane S, Vaidya A, Dhimal M, Dhakal P, et al. (2015) The Burden and Determinants of Non Communicable Diseases Risk Factors in Nepal: Findings from a Nationwide STEPS Survey. PLoS ONE 10(8): e0134834. <https://doi.org/10.1371/journal.pone.0134834>
ii. Bista B, Mehata S, Aryal KK, Thapa P, Pandey AR, Pandit A, et al. Socio-demographic Predictors 518 of Tobacco Use among Women of Nepal: Evidence from Non Communicable Disease Risk Factors STEPS 519 Survey Nepal 2013. Journal of Nepal Health Research Council. 2015;13(29):14-9. Epub 2015/09/29. 520 PubMed PMID: 26411707.

Even then this work does not lose its merit but the title should change evidence of clustering from this work may be misleading because of missing variables (3 out of 8). Hence whilst clustering may remain in the body of the work as one of the objectives of the study it should be removed from the title

iii. Olawuyi AT, Adeoye IA (2018) The prevalence and associated factors of noncommunicable disease risk factors among civil servants in Ibadan, Nigeria. PLoS ONE 13(9): e0203587.https://doi.org/10.1371/journal. pone.0203587

I suggest the new title should be simply “**Socio-demographic correlates of selected Non-Communicable Diseases risk factors among reproductive aged women of Nepal: Results from Nepal Demographic Health Survey 2016”**

However, there is need to justify additional evidence above that which has been provided by Aryal and his co-workers. In addition, authors need to emphasise the importance of NCD risk factors among women of reproductive age. Which may be an important contribution of their study.

2. The authors need to employ an English editor – The flow of the written is still poor. For instance, this section in the result could start with the mean age and standard deviation. The description of the table should flow sequentially

*Just over half (53.95%) of the participants were d15-29 years. Largest proportions (36.62%) of the participants were from Janjati group (indigenous group). One thirds (33.34%) had no formal schooling while 76.655% of the participants were married. Most of the participants belonged to the Terai belt (49.89%) and rural areas (63.30%). Similarly, 22.43% and 20.92% of participants belonged to richer and the richest wealth quintile. Most of the participants were engaged in agriculture or were self-employed*

3. There a need for a ROBUST description of the study area and setting.

The Provinces – their characteristic features, level of development including infrastructure, are rural or urban, level of westernization and epidemiologic/ nutritional transition going on. This will make interpretation and discussion of results more meaningful. This should apply to the ecological region and ethnic group. For example, it is not clear why all the other provinces (Provinces 1 – 5, Karnali etc should have a lower risk for smoking, overweight and hypertension compared to Province 1)

4. DISCUSSION still needs to be worked on

*“Our study demonstrated that the proportion of tobacco use was nearly 3 fold higher in 30-40 years age group women which has also been observed in previous studies [23, 24].”*

We need to know why tobacco use is higher among older women of reproductive age compared to younger women, what does this imply and what are your recommendations. Not sufficient to state that it *has also been observed in previous studies [23, 24].”*

5. NEED TO WRITE A STRONG LIMITATION SECTION – in the light of limited variables
6. OTHERS
   a. Variable definitions
      i. Is it overweight or overweight and obesity
      ii. Occupational status . The categories not seem homogenous Agriculture does that mean self employed? What is services?

Generally, authors need to follow the systematic reporting of Plos one articles. There are several articles published on NCD risk factors

Ikeola Adeoye
